# Pleistocene allopatric differentiation followed by recent range expansion explains the distribution and molecular diversity of two congeneric crustacean species in the Palaearctic

**DOI:** 10.1038/s41598-021-02164-8

**Published:** 2021-11-24

**Authors:** Dunja Lukić, Tom Pinceel, Federico Marrone, Monika Mioduchowska, Csaba F. Vad, Luc Brendonck, Robert Ptacnik, Zsófia Horváth

**Affiliations:** 1 WasserCluster Lunz, Lunz am See, Austria; 2 Research Department for Limnology, University of Innsbruck, Mondsee, Austria; 3 Laboratory of Animal Ecology, Global Change and Sustainable Development, KU Leuven, Leuven, Belgium; 4 Community Ecology Laboratory, Department of Biology, Vrije Universiteit Brussel (VUB), Brussels, Belgium; 5 Centre for Environmental Management, University of the Free State, Bloemfontein, South Africa; 6 Department of Biological, Chemical and Pharmaceutical Sciences, University of Palermo, Palermo, Italy; 7 Department of Genetics and Biosystematics, University of Gdańsk, Gdańsk, Poland; 8 Department of Marine Plankton Research, University of Gdańsk, Gdynia, Poland; 9 Institute of Aquatic Ecology, Centre for Ecological Research, Budapest, Hungary; 10 Laboratory of Aquatic Ecology, Evolution and Conservation, KU Leuven, Leuven, Belgium; 11 Water Research Group, Unit for Environmental Sciences and Management, North-West University, Potchefstroom, South Africa; 12 Department of Invertebrate Zoology and Hydrobiology, Faculty of Biology and Environmental Protection, University of Lodz, Banacha 12/16, 90-237 Lodz, Poland

**Keywords:** Phylogenetics, Biodiversity, Biogeography

## Abstract

Pleistocene glaciations had a tremendous impact on the biota across the Palaearctic, resulting in strong phylogeographic signals of range contraction and rapid postglacial recolonization of the deglaciated areas. Here, we explore the diversity patterns and history of two sibling species of passively dispersing taxa typical of temporary ponds, fairy shrimps (Anostraca). We combine mitochondrial (COI) and nuclear (ITS2 and 18S) markers to conduct a range-wide phylogeographic study including 56 populations of *Branchinecta ferox* and *Branchinecta orientalis* in the Palaearctic. Specifically, we investigate whether their largely overlapping ranges in Europe resulted from allopatric differentiation in separate glacial refugia followed by a secondary contact and reconstruct their postglacial recolonization from the inhabited refugia. Our results suggest the existence of distinct refugia for the two species, with genetic divergence among intraspecific lineages consistent with late Pleistocene glacial cycles. While *B. ferox* lineages originated from Mediterranean refugia, the origin of *B. orientalis* lineages was possibly located on the Pannonian Plain. We showed that most dispersal events predominantly happened within 100 km, coupled with several recent long-distance events (> 1000 km). Hence the regional habitat density of suitable habitats in Central Europe is possibly a key to the co-existence of the two species. Overall, our study illustrates how isolation in combination with stochastic effects linked to glacial periods are important drivers of the allopatric differentiation of Palaearctic taxa.

## Introduction

The Pleistocene epoch (2.5 mya—11 kya) was characterised by extreme climate fluctuations including repeated cold periods with widely extended ice cover and milder periods of glacial retreat^1^. In temperate areas, glaciation periods were associated with local population extinctions, southward range shifts (in the northern hemisphere) and resulting genetic bottlenecks^2,3^. Conversely, milder periods were associated with rapid northward range expansions^2^. These historic climatic fluctuations and the associated range shifts left an imprint on the contemporary distribution and genetic diversity of several taxa in the temperate zone^3,4^. Recent research also revealed that the current distribution patterns of populations and closely related taxa are associated with species traits including those related to dispersal^5,6^. Still, the processes of differentiation and global patterns of gene flow remain largely unexplored in many organisms and ecological groups.

The increasing application of molecular methods and rising number of phylogeographic studies in the last couple of decades facilitated the identification of the most important Pleistocene refugia across the Palaearctic (e.g. see Hewitt^2,7^). In Europe, the Balkan, Apennine, and Iberian Peninsulas had the mildest climate during glaciations and acted as important refugia for many temperate species^3,7,8^. Consequently, these regions nowadays host high genetic diversity and local endemics^8^, a recurrent pattern also formulated as the “northern purity vs. southern richness paradigm”^2,9,10^. In addition, North Africa^11^ and the Middle East and its surrounding regions are also identified as possible refugia for several European taxa^12,13^.

Recently, an increasing number of phylogeographic studies stressed the importance of regions often referred to as ‘cryptic refugia’^14–16^. Cryptic refugia are individual regions located further north from classical refugia, and are believed to have maintained a suitable and stable environment for the survival of temperate species during glaciations^14,17^. These regions may have acted as important hubs from where many species could have expanded their range after the glacial retreat faster than from the southern peninsulas behind geographical barriers, such as mountain systems and the Mediterranean Sea^14^. One such extra-Mediterranean example is the Pannonian Plain in Central Europe, which likely acted as a cryptic refugium for several groups, e.g. crustaceans, amphibians, fish and mammals^16,18–22^.

Each of the Pleistocene refugia could have featured specific environmental conditions and biotic interactions that forced populations of individual species to adapt locally. Together with the increased importance of drift in isolation^23^, this has led to genetic differentiation among populations creating the opportunities for allopatric differentiation processes resulting in intraspecific differentiation and sometimes even speciation^2,8^. After the glacial retreat, these newly formed evolutionary units expanded, which led to co-occurrences between sister lineages and species^9,24^. Several of these genetic lineages are morphologically cryptic, while still representing unique evolutionary potential (i.e. evolutionary significant unit) and should thus be protected to preserve the adaptive capacity of species^25–27^. The current biodiversity crisis, with its severe environmental changes and high rates of extinction, calls for a better understanding of this genetic diversity, together with the functioning of habitat networks and genetic connectivity therein.

While terrestrial species could spread relatively fast after the last glacial retreat, quickly filling the new continuous territories opening up in the temperate regions of Europe^7^, these post-glacial expansion scenarios may have been fundamentally different for aquatic species inhabiting lentic freshwater ecosystems, such as ponds and lakes. The discrete nature and patchy distribution of these habitats are major constraints for dispersal in general^28^. Given that the movements of these taxa in a landscape depend on the availability of suitable vectors such as wind^29,30^ or animals^5,31–33^, their re-colonization should be very constrained or stochastic in patchy habitat networks. The so-called “large branchiopods” (including five extant orders: Anostraca, Notostraca, Spinicaudata, Laevicaudata and Cyclestherida) are well adapted and almost exclusively limited to temporary ponds^34,35^. They have long been considered as a flagship group for temporary ponds and their conservation^36^. Their functional importance, such as community-shaping (anostracans and notostracans^37–40^) and ecosystem engineering (notostracans^40^) is also increasingly recognized. They produce resting eggs to bridge the unfavourable parts of the year in their local habitats. Their resting eggs can remain viable buried in the sediment for years before hatching^41,42^ and play an important role in the passive dispersal of this group.

**Figure 1:**
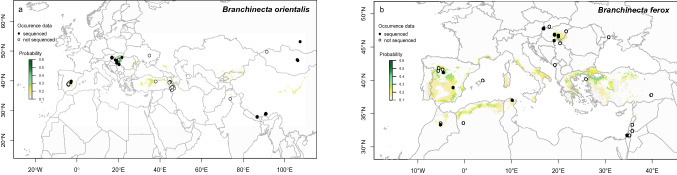
(**a**) The currently known distribution of the two study species, *Branchinecta ferox* (**a**) and *Branchinecta orientalis* (**b**), based on published distribution data in the last 50 years (circles), the populations included in the molecular analyses (filled circles), and their predicted distribution area (yellow to green shading) according to bioclimatic data. Maps were created with the ‘dismo’ package^101^ of R v. 4.0.3^102^.

Here, we analyse the genetic diversity of two congeneric anostracan species (Crustacea, Branchiopoda) belonging to the ancient genus *Branchinecta*, which inhabit temporary ponds across the Palaearctic. We apply a comprehensive approach that involves sequencing of both mitochondrial and nuclear DNA regions of around 200 specimens from 56 populations (Fig. 1). First, our aim is to determine whether these two species survived the Pleistocene glaciations in southern refugia, similar to most pond-inhabiting species^43–45^. Second, we want to shed light on the natural history of these closely related species, and specifically look for signs of allopatric differentiation in separate refugia. Third, we aim to determine post-glacial (re)colonization patterns and discuss the importance of potential biotic or abiotic vectors that mediated the passive dispersal of the studied anostracan species. The discontinuous (island-like) distribution of *Branchinecta ferox* and *Branchinecta orientalis* populations increases the potential for their genetic isolation and differentiation^46^. Consequently, we expect to find a rather high genetic variation in both species on a global scale, especially in geographically isolated regions, while we expect that populations along the main migration routes of water birds (such as North Africa, Iberian Peninsula and Central Europe) should share common haplotypes.

## Results

### Genetic diversity

Based on the mitochondrial COI DNA region, the mean interspecific divergence between *B. ferox* and *B. orientalis* was 10.1% (Table 1). For *B. ferox*, the overall mean intraspecific genetic variation was 4.0% (Table 1) based on 13 identified haplotypes. One haplotype was relatively common across Central Europe and was found in 11 populations, while others were mostly present in single populations. The highest genetic divergence (8.4%) was found between a Moroccan specimen (M1) and another from Israel (IS1). For *B. orientalis*, the overall mean intraspecific genetic variation was 2.9% (Table 1) based on 22 haplotypes. Two haplotypes were relatively common throughout Central Europe, with one present in 13 and another in 10 populations. Other haplotypes were only recorded from one to three populations each. The highest pairwise distance (5.8%) was found between one Hungarian specimen (HK4) and individuals from two populations in Austria (AP1, AR4).

**Table 1:** Kimura 2-parameter genetic distances with partial deletion of 90% for both *Branchinecta* species and two genetic markers, mitochondrial CO1 and nuclear ITS2 DNA regions.

	Generated sequence length (bp)	No of generated sequences	No of sequences included*	No of populations included	No of identified haplotypes	K2P genetic distances		
						Min	Mean	Max
**Mitochondrial CO1 DNA region**								
*Branchinecta ferox*	538–658	34	42	16	13	0.2	4.0	8.4
*Branchinecta orientalis*	443–658	73	91	31	22	0.2	2.9	5.8
*Branchinecta ferox* and *B. orientalis*	443–658	107	133	/	/	8.5	10.1	12.7
**Nuclear ITS2 DNA region**								
*Branchinecta ferox*	407–626	25	32	15	3	0.9	3.7	4.8
*Branchinecta orientalis*	477–612	61	73	35	7	0.2	1.5	2.7
*Branchinecta ferox* and *B. orientalis*	407–626	86	105	/	/	10.1	11.5	14.3

*Additional sequences were downloaded from GenBank (MW829405-MW829407^47^, LT821325-LT821341^89^ and LC469606^96^). For more details on produced sequences and studied populations, see Table A1 in the Appendix B.

Based on the nuclear ITS2 DNA region, the mean interspecific distance between *B. ferox* and *B. orientalis* was 11.5% (Table 1). For *B. ferox*, the overall mean intraspecific genetic differentiation was 3.7% (Table 1) considering 3 haplotypes. One haplotype was widely distributed across Central Europe and present in Spain and Morocco. For *B. orientalis*, the overall mean intraspecific genetic differentiation was 1.5% (Table 1) considering 7 haplotypes. Three (out of four) haplotypes in Central Europe were rare and each was present in only one population.

For the nuclear 18S region, we generated 16 sequences (five sequences of *B. ferox* and 11 sequences of *B. orientalis*) of 598 to 1719 bp. We added two further *B. orientalis* sequences from China to the set of sequences^47^. We found only one mutation (C or T) between the two examined *Branchinecta* species on the nuclear 18S region. No variability was observed within *B. ferox*. In *B. orientalis*, we found genetic variation at only two bases between the Mongolian and all other specimens. The existence of one more haplotype identified by Deng et al.^47^ is hence possibly a misinterpretation that occurred via a mistake in generating the sequence MW829399, as there is a T nucleotide insertion close to the end of the sequence.

### Phylogenetic analyses

**Figure 2:**
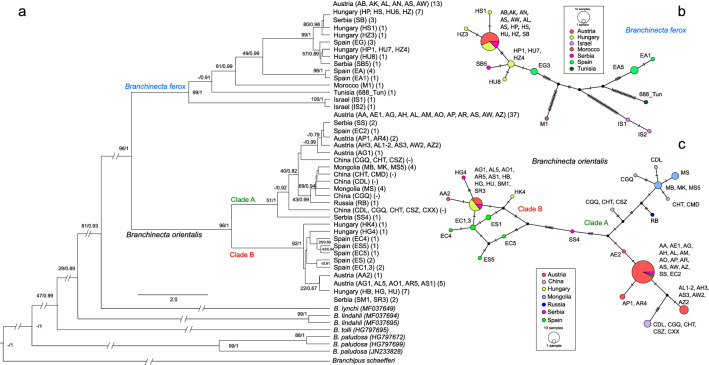
(**a**) Phylogenetic tree of *Branchinecta ferox* and *B. orientalis* along with other *Branchinecta* species based on the mitochondrial COI gene fragment. The supporting values based on two models (maximum likelihood/Bayesian inference) are included close to the nodes. Localities are specified in the first and the number of specimens in the second brackets. The unsupported groupings are indicated with ‘-‘. (**b**) Haplotype network of *B. ferox* based on the median joining network. Black circles represent missing haplotypes. Short vertical bars indicate the number of mutations between haplotypes. The haplotype network analysis identified 68 segregating sites, of which 43 were recognized as parsimony informative. (**c**) Haplotype network of *B. orientalis* based on the median joining network. The haplotype network analysis identified 33 segregating sites, of which 21 were recognized as parsimony informative.

At mitochondrial COI DNA region, both methods of phylogenetic inference (maximum likelihood [ML] and Bayesian inference [BI]) produced trees with similar topologies. *B. ferox* (Fig. 2a, b) populations can be subdivided into five main haplogroups, corresponding to distinct geographical regions. These haplogroups represent the Middle East, two regions in Northern Africa (Tunisia vs. Morocco) and two in Europe (Spain vs. Spain and Central Europe). One group in Europe included specimens from the Albacete region in south-east Spain and the second included populations from Segovia in central Spain and all populations from Central Europe. For *B. orientalis* (Fig. 2a, c), the phylogenetic search methods (ML and BI) grouped the studied *B. orientalis* haplotypes in two larger haplogroups (Clade A & B). Populations from Central Europe were present in both haplogroups. Most of the Spanish individuals (except for one specimen from Cuenca region, east-central Spain) belonged to Clade B.

Both phylogenetic reconstructions of nuclear ITS2 DNA region first differentiated *B. ferox* from *B. orientalis* species (Fig. A1 in Appendix C). Within *B. ferox*, it was possible to separate three groups, one in the Middle East (Israel), another in the Iberian Peninsula (Albacete), and a third from North Africa (Morocco), the Iberian Peninsula (Segovia) and Central Europe. Unfortunately, no ITS2 sequences were obtained from the Tunisian population of the species.

For *B. orientalis*, reconstructions based on the ITS2 region suggested slightly different phylogenetic relationships compared to the COI DNA sequences. The sequences from Spain and both Mongolia and China were grouped together but were further subdivided into two distinctive haplogroups matching the geographic origin of the samples. Except for the two sequences originating from two populations in Austria and Serbia (AW1 and SO4) that formed their own haplogroup, all populations from Central Europe belonged to another haplogroup.

### Genetic and spatial distance based on the mitochondrial COI gene region

**Figure 3:**
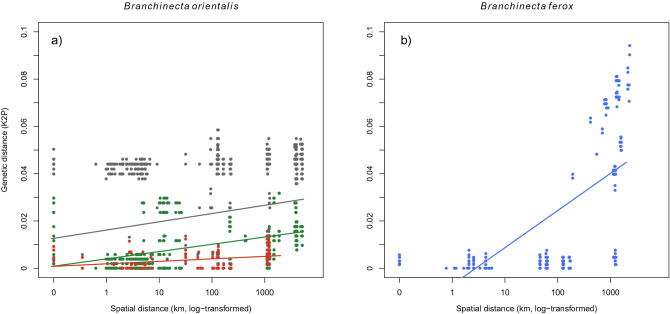
Genetic differentiation (genetic distances measured as Kimura 2-parameter; K2P) in relation to spatial distance, based on the mitochondrial COI gene fragment. (**a**) In *B. orientalis*, a significant distance decay relationship was found both for the full dataset (grey; Mantel test; nperm = 999, r = 0.22, *p* = 0.001) and in the separate analyses of the two clades (green: Clade A; r = 0.53, *p* = 0.001, red: Clade B; r = 0.26, *p* = 0.001; for the clades, see Fig. 2a,c). (**b**) In *B. ferox*, there was a significant distance decay in the full dataset (blue; r = 0.65, *p* = 0.001).

A significant distance-decay relationship was revealed for pairwise genetic distances (K2P) of both *B. orientalis* (Fig. 3a; Mantel test: r = 0.22, *p* = 0.001) and *B. ferox* (Fig. 3b; Mantel test: r = 0.65, *p* = 0.001). This was also significant in the two clades (Clades A and B of *B. orientalis*) when tested separately (Fig. 3a). In Clade A, which included populations from the Russian Federation, China, and Mongolia, we found a stronger relationship with spatial distance (r = 0.53, *p* = 0.001) than in the entire European Clade B (r = 0.26, *p* = 0.001).

**Figure 4:**
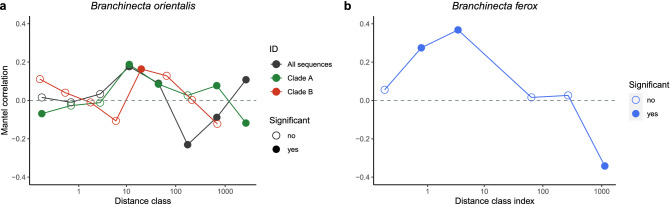
Mantel correlogram for genetic distance (Hellinger-transformed) and geographic distance (log-transformed) in (**a**) *Branchinecta orientalis* (black – all sequences; green – Clade A; red – Clade B; for the clades, see Fig. 2a,c) and (**b**) *Branchinecta ferox* based on the mitochondrial COI gene fragment. The circles indicate significant correlation within a distance class, tested within eight distance classes in each of the three datasets of *B. orientalis* and seven distance classes in *B. ferox*.

In agreement with the steeper slope of distance-decay in *B. ferox*, the signs of autocorrelation were also stronger in this species based on Mantel correlations. Here, we found positive autocorrelation between genetic distances and spatial distances within 100 km (Fig. 4b), which turned negative over larger spatial scales. A similar trend was found in *B. orientalis*, with most positive relationships within 100 km (Fig. 4a), but in the entire dataset as well as in Clade A, we found two cases of significant positive correlation even on the larger scale (> 100 km), indicating successful long-distance dispersal events.

## Discussion

Our study illustrates how historical long-term isolation can have a lasting impact on the current distribution and genetic diversity of sibling species. Even though the present distribution of the congeneric anostracans *Branchinecta ferox* and *B. orientalis* overlap substantially within Europe, our results suggest that this was not the case during the Pleistocene. Moreover, our data on current regional genetic diversities provided evidence for the existence of multiple Pleistocene refugial regions for both species. This has resulted in distinct and well-characterised evolutionary lineages within the two species, both through new mutations and lineage sorting acting on their pre-existing genetic variability, revealing a history of allopatric differentiation. Based on haplotypes shared between geographically distant regions, we could detect several recent long-distance dispersal events (> 1000 km). At the same time, our results based on population similarities implied that most dispersal events predominantly happen on a much smaller spatial scale (within 100 km). This is in good agreement with the growing evidence that the realized dispersal in passively dispersing large branchiopods is routinely occurring at the scale of few tens of km, with higher values in arid areas and open grasslands, and lower values for the species occurring in forested areas (e.g.^48–50^).

In our study, we applied multiple molecular markers to unravel the species history of the studied sibling species of *Branchinecta*: one mitochondrial (COI) and two nuclear (ITS2 and 18S). They generally point to the allopatric speciation of the two species, as well as to the reason behind their intraspecific differentiation: their survival in different Palearctic refugia during the Pleistocene. Applying the evolutionary rates for COI^51^, the most recent common ancestor of the two species is to be located at around 5.69 mya, i.e. in the late Miocene or in the Pliocene. In *B. ferox* populations, differentiation likely started earlier than in *B. orientalis*, as the North African and the Middle East clades themselves are well differentiated from each other (around 8% distance on COI region and 5% on ITS2 region), and the major clades were separable based on both the COI and ITS2 DNA regions. All COI haplotypes in *B. orientalis* were classified into two haplogroups, while in the ITS2 tree, divergence was generally low. The slightly different tree topologies found for these two markers (especially in Central Europe, that holds a large number of local populations of *B. orientalis*) could be explained by a relatively recent split between the two clades combined with the different inheritance mechanisms: while mitochondrial genes are inherited from the mothers (but see exceptions to that rule in Lindholm et al.^51^), nuclear genes are typically inherited from both parents.

As we found generally higher phylogeographic diversity (both based on COI and ITS2 DNA) of *B. ferox* in the southern parts of its current distribution range, this indicated multiple potential refugia in this region (i.e., Northern Africa, the Middle East, and possibly Spain). The Mediterranean areas of Africa and Asia were not covered by ice during the Pleistocene and provided suitable habitats to serve as refugia for many species^8,11,24^ including temporary pond-dwellers, e.g., copepods^10^ and other anostracan species^43^. From the southern Mediterranean region, *B. ferox* possibly (re)colonized the Iberian Peninsula first as supported by the presence of two haplogroups for both genetic markers (Fig. 2a & Fig. A1). Moreover, the (re)colonization of the Iberian Peninsula (i.e., Europe) might have started long before the last glacial maximum. Another possibility is that *B. ferox* was present in the Iberian Peninsula throughout the Pleistocene in which case the Iberian Peninsula would have acted as another Pleistocene refugium for *B. ferox*.

For *B. orientalis*, our phylogeographic data suggest a Pleistocene refugium located in the Pannonian Plain or in a nearby region, e.g., the Balkan Peninsula, which is a much more common refugium for several species; however, given that *B. orientalis* currently only occurs in the Pannonian Plain, we suggest this as the more likely scenario. This hypothesis is supported by the relatively high diversity observed on both genetic markers in Central Europe and by the topology of the constructed haplotype network. The presence of two well-differentiated COI clades on the Pannonian Plain indicates the existence of two separated populations—possibly due to a “refugia within refugium” pattern^24^—during the glaciation periods. Based on the COI gene region, we can assume that all current *B. orientalis* populations originated from the Pannonian Plain (and/or the Balkan Peninsula); it implies that colonization of the Iberian Peninsula happened through at least two distinct events of colonization. This is suggested by the fact that on the Iberian Peninsula all samples except for one specimen belonged to one of two identified COI haplogroups. In this case, the re-colonization route we revealed from the Pannonian Plain to the Iberian Peninsula is quite unique for Palaearctic species in general: all other taxa that had a refugium in the Pannonian Plain during the Pleistocene have a much more restricted range today (e.g. amphibians^21,52^). It is also possible that the colonization of the Iberian Peninsula and Central Asia happened during one of the previous interglacial periods (as also suggested for cladocerans^53^). In particular, most populations in Central Asia constitute a separate (sub)clade based on the sequenced COI and ITS2 regions and some even show minor differences on the 18S region in comparison to the other populations of *B. orientalis*, which supports this possibility.

Our genetic data showed strong bottleneck effects of the Pleistocene glaciations in both species. In *B. ferox*, there was a very clear spatial structure visible on the phylogenetic tree, and it was even more explicitly shown in the spatial analyses in *B. orientalis* and *B. ferox* (based on both distance decay and spatial autocorrelation analyses). Aquatic passive dispersers often show clear geographic structuring (e.g. in *Branchipus schaefferi*^43^ and *Triops cancriformis*^54^), which is also the case with the two studied *Branchinecta* species (see Figs. 3 and 4). At the same time, there can be marked differences in the prevalence of long-distance dispersal events between the species with distinct habitat requirements. For species occupying smaller ephemeral habitats, wind can be a more important dispersal vector^55^, with dispersal events mostly happening within very short distances^29,30,56,57^. Such species include most anostracans (e.g., *Branchipodopsis wolfi*^56^ and notostracans (*Triops cancriformis*^54^), where indeed there is no or very scarce indication for long-distance dispersal events. The upper end of this “mobility” gradient is the anostracan genus *Artemia* frequently inhabiting larger waterbodies: here long-distance dispersal by waterbirds feeding on *Artemia*^58^ (and even by humans^59^) leads to gene flow detectable even at larger scales^60,61^. Our study species are somewhere between these two extremes, with visible geographic structuring but with several possible long-distance dispersal events visible in the data (e.g., several extremely low values of genetic distance over 1000 km in *B. orientalis* or the Spanish *B. ferox* population clustering together with populations from the Pannonian Plain).

Here, we found that most dispersal events in both *Branchinecta* species have been happening within 100 km. This distance is well in accordance with the dispersal habits of most waterbirds^62,63^. The role of waterbirds as dispersal agents is well documented for many aquatic invertebrates, including anostracans^31,62,64–66^. The studied *Branchinecta* species in Central Europe and Spain inhabit shallow sodic lakes of a relatively large surface area^67,68^, situated along the seasonal migration routes of a diverse set of waterbird species^69^, among which several are even proven to be attracted to habitats with the most abundant *Branchinecta* populations^70^. This altogether implies the dominant role of waterbirds connecting local populations. Right after the glacial retreat, the dispersal of *Branchinecta* could have been further facilitated by historic long-distance mammal migrations, specifically related to the extinct megafauna^71^, e.g. mammoth species (*Mammuthus* spp.), that are known to carry diverse propagules including *Branchinecta* resting eggs on their body^72^. They once inhabited a vast area of Eurasia and moved over large distances^73,74^, similar to their extant sibling species the African elephants, also known as vectors for passive dispersers^75^. However, nowadays large mammals can only contribute to small-scale dispersal events^33,76^.

Finally, the high genetic diversity of *B. orientalis* in eastern Austria underlines the importance of dense habitat networks for maintaining both local and regional genetic diversity, which is further supported by our results showing high dispersal rates within smaller regions (< 100 km). This calls for adequate protection of the inhabitants of temporary aquatic systems on the regional (metapopulation) level, together with their aquatic habitats. Moreover, particular attention should be paid to the long-term conservation of the patchily distributed and isolated populations of *B. ferox* in Africa and Asia, which host unique and well-differentiated endemic lineages currently at risk of extinction. The realisation of conservation programmes aimed at the long-term persistence of these significant evolutionary units is strongly desirable.

## Methods

### Study species

The anostracan genus *Branchinecta* comprises around 50 species. Its members are found on all continents except Australia^77–79^. The genus is represented by five species in the Palaearctic, with only two being present in the study area: *Branchinecta ferox* and *Branchinecta orientalis*. *B. orientalis* inhabits mineral-rich temporary waters and has a disjunct distribution ranging between 27° and 55° N in Europe and Asia (Fig. 1a). Active populations generally occur in spring, but they have also been recorded in autumn or winter^80–82^. *B. ferox* has a circum-Mediterranean and Central European distribution (Fig. 1b). It is the only *Branchinecta* species occurring in Africa, being present in the north-western part of the continent (Morocco, Algeria and Tunisia^78^). In Europe, it occurs in Spain and Central Europe (Pannonian Plain), and its range extends further east across South Ukraine to the west of Russia^83,84^. This species has also been reported in the Middle-East (i.e. Jordan, Israel and Turkey^85–88^). *B. ferox* is a halotolerant species, occurring both in freshwater rain pools in the circum-Mediterranean area^85^ and saline pans in the Pannonian Plain^68^. Active populations mostly occur in late winter and early spring^68,78,81^. The geographic distribution of these two *Branchinecta* species overlaps in the Pannonian Plain, Iberian Peninsula and Turkey^68,87,89^. On the Iberian Peninsula and the Pannonian Plain, the two species are found almost exclusively in large and shallow saline pans, and represent a preferred food source for waterbirds on their seasonal migration routes^70^.

### Species distribution maps

We compiled a list of known occurrences of both species based on the above listed samples and literature data^47,67,68,78,83–87,89–100^. The literature sources mentioning distribution and ecology of *B. ferox* and *B. orientalis* were searched via Google Scholar and Web of Science. Sources that did not report precise habitat coordinates of populations and/or are older than 50 years are not included, hence the actual distribution of the species is probably underrepresented (e.g., the actual distribution of *B. orientalis* in Asia is most likely underrepresented here). To account for this, we built species distribution maps with the ‘dismo’ package^101^ of R v. 4.0.3^102^. Here, we used all available bioclimatic variables from the WorldClim database (http://www.worldclim.org)^103^, and predicted the probability of occurrence for each species. Although these variables do not include the presence of suitable habitats (i.e., shallow temporary waters, for which there is no publicly available database yet), they should provide a reliable indication for the climatic conditions where suitable habitats are likely to occur. According to the probability maps, our general coverage of sequenced samples was in a good agreement with the overall distribution of both species, including samples from the Mediterranean, the Pannonian Plain in Central Europe (both species), and Middle to Central Asia (*B. orientalis*). Even though our model predicted the possible occurrence of *B. ferox* in Italy and Southern France (Fig. 1b), we can mostly exclude these latter regions given that both are very well covered by previous Anostraca studies that have never reported the species there^104,105^.

### Sampling procedure

We collected *Branchinecta orientalis* specimens from 29 temporary pools, ponds and shallow lakes in Europe and Asia (Table A1). *Branchinecta ferox* specimens were collected from 16 habitats in Europe, North Africa, and Asia (Table A1). Specimens were collected between 1971 and 2018 and fixed in ethanol (of various concentrations). Once the samples arrived at the lab, animals were transferred immediately to pure ethanol until further processing. All specimens were dissected to obtain phyllopod tissue for DNA extraction. For the molecular laboratory procedures to acquire the DNA sequences for the targeted gene regions, see Appendix B.

### Reconstructions of phylogeny based on mitochondrial COI and nuclear ITS2 DNA region

All generated *B. ferox and B. orientalis* sequences were assembled and visually checked for quality in SeqScape v3. We checked the COI alignment for indels and internal stop codons that would indicate unintentional amplification of nuclear pseudogenes^106^. The produced sequences were edited in BioEdit^107^. The newly produced sequences were aligned together with the existing sequences in GenBank (for *B. ferox* and *B. orientalis* see Table A1 in Appendix 1A; *Branchinecta lynchi* MF037649; *B. lindahli* MF037694-5; *B. tolli* HG797695; *B. paludosa* HG797672, HG797699 and JN233828)^47,51,89,96,108,109^ and one outgroup taxon (for COI, we used *Branchipus schaefferi* MK449416^43^ and for ITS2, *Chirocephalus diaphanus* LT860206^89^) by using CLUSTALW multiple alignment tool in BioEdit for the CO1 gene region and MUSCLE for the ITS2 DNA region. The most likely evolutionary model for the COI marker was determined in in PartitionFinder2^110^ and for the ITS2 in MEGA X^111^ based on the Akaike Information Criterion (AIC). For the COI gene region, the AIC selected a General Time Reversible model (GTR), which was used to reconstruct ML and BI tree. For the ITS2 DNA region, the AIC selected for GTR model with a gamma shape parameter (+ G, γ = 1.22), which was used to reconstruct ML and BI tree.

ML analyses were performed in MEGA X with 1000 bootstrap replicates. Bayesian inference was performed in BEAST v2.6.4^112^ in case of the COI gene region. The settings included the strict molecular clock, Yule model and a lognormal prior distribution for the taxon set of the *Branchinecta paludosa* samples (set as monophyletic; mean ± standard deviation: 1.25 ± 0.15 as in Lindholm et al.^51^). The analyses were run for 10 million generations. Molecular evolutionary rates of 2% divergence per million years were applied by Lindholm et al.^51^ on the closely related *B. paludosa*, and were thus here applied to get a tentative temporal frame for the main cladogenetic events observed within our study taxa. We used TreeAnnotator v. 2.6.4 to construct a single tree by discarding 25% of the compiled trees as a burn-in. As molecular clock is not available for the ITS2 DNA region, we used MrBayes^113–115^ to an ITS2 phylogenetic tree using BI. We applied the Markov Chain Monte Carlo (MCMC) method for 10^6^ generations (standard deviation of split frequencies reached < 0.01) while the trees were sampled every 1000 generations. The initial 25% of produced trees were discarded as burn-in.

For the *B. ferox* and *B. orientalis* COI gene fragments, we built a median-joining haplotype network for each species (ε = 0; Bandelt et al., 1999) using PopART v 1.7^117^; http://popart.otago.ac.nz). The sites containing missing bases at the end and the beginning of the alignment, as well as ambiguous bases, were masked leaving 479 (*B. ferox*) and 304 (*B. orientalis*) sites for further network analysis.

### Analysis of genetic diversity

Substitution saturation was tested in DAMBE v. 7.0.28^118^, using the default settings and including all sites. The index of substitution saturation (Iss) was significantly smaller than the critical index of substitution saturation (Iss c), indicating little saturation^119,120^ for both markers. Pairwise genetic K2P distances between all generated sequences and the mean genetic distances within and among the main groups in the phylogeny of *B. ferox and B. orientalis* were calculated in MEGA X^121^ with partial deletion of 90% (515 positions in the final data set for COI and 574 positions for ITS2). The haplotype number was determined in DnaSP 6^122^.

In both *B. ferox* and *B. orientalis*, we tested for the dispersal limitation based on the relationship between pairwise genetic differences on the mitochondrial COI gene region and geographic distances. To do so, we exported pairwise genetic distances from MEGA X in a form of a data matrix and applied Hellinger transformation. We calculated pairwise geographic distances between all sampling sites as orthodromic distance. To reveal effective dispersal over distinct distance classes, we used the computed pairwise genetic distances and log + 0.1 transformed spatial distances to perform a Mantel test with 999 permutations and calculate Mantel correlation coefficients. In addition to the full dataset, separate Mantel tests were performed within two main *B. orientalis* clades (Clade A and Clade B). Mantel correlation coefficients were calculated between pairwise genetic distances within eight distance classes for all COI sequences of *B. orientalis* and repeated separately for the two main clades to detect positive autocorrelation as signs of effective dispersal. For *B. ferox*, we calculated Mantel correlation coefficients between pairwise genetic distances within seven distance classes as the highest spatial distance between *B. ferox* populations was lower than between individual *B. orientalis* populations. Calculation of pairwise spatial distances, Mantel tests and Mantel correlation coefficients were performed in R software, with the ‘fields’^123^ and ‘vegan’^124^ packages.

## Supplementary Information

Supplementary Information.

## Data Availability

The DNA sequence data supporting the findings of this study are openly available in GenBank at https://www.ncbi.nlm.nih.gov/genbank/, accession numbers are listed in the Appendix A, Table A1.
