# Supplementary material for: Pleistocene allopatric differentiation followed by recent range expansion explains the distribution and molecular diversity of two congeneric crustacean species in the Palaearctic
Source: Sci Rep. 2021 Nov 24;11:22866. doi: 10.1038/s41598-021-02164-8 (PMC8613293; doi:10.1038/s41598-021-02164-8)
Supplement: Supplementary file 1 — Supplementary Information. [file 41598_2021_2164_MOESM1_ESM.docx]

Pleistocene allopatric differentiation followed by recent range expansion explains the distribution and molecular diversity of two congeneric crustacean species in the Palaearctic

Dunja Lukić, Tom Pinceel, Federico Marrone, Monika Mioduchowska, Csaba F. Vad, Luc Brendonck, Robert Ptacnik & Zsófia Horváth

Appendix A – Methods: Sampling procedure

Table A1. Overview of all specimens of *Branchinecta ferox* and *Branchinecta orientalis* used in the analyses, with details on the sequence IDs *(remaining* *COI and 18S sequence numbers available upon revision*), collection localities and number of sequenced specimens (in brackets). **In bold** - sequences from other sources (from China ^1^; the Russian Federation ^2^; and in Hungary and Spain ^3^).

| **Nr.** | **ID** | **Country** | | **Region, locality** | **Latitude** | **Longitude** | | **COI sequence ID** | | **ITS2 sequence ID** | **18S sequence ID** |
| --- | --- | --- | --- | --- | --- | --- | --- | --- | --- | --- | --- |
| ***Branchinecta ferox*** | | |  | |  |  | |  | |  |  |
| 1 | AB1 | Austria | | Apetlon, Birnbaumlacke | 47.817715 | 16.864911 | | OK047162 (1) | | MW488064 (1) | - |
| 2 | AN1-2 | Austria | | Apetlon, Kühbrunnlacke | 47.792736 | 16.878626 | | OK047163-4 (2) | | MW488065-6 (2) | - |
| 3 | AL6 | Austria | | Apetlon, Lange Lacke | 47.757475 | 16.878765 | | OK047165 (1) | | - | - |
| 4 | AS6-7 | Austria | | Apetlon, Sechsmahdlacke | 47.783789 | 16.884112 | | OK047166-7 (2) | | - | OK037203 (1) |
| 5 | AW6 | Austria | | Apetlon, West. Wörthenlacke | 47.770922 | 16.870777 | | OK047168 (1) | | MW488067 (1) | OK037205 (1) |
| 6 | AK5-10 | Austria | | Illmitz, Kirchsee | 47.758679 | 16.785489 | | OK047169-74 (6) | | MW488068-71 (4) | OK037197-8 (2) |
| 7 | HO1-2 | Hungary | | Csongrád, Kis-sóstó | 46.74096 | 19.99218 | | - | | MW488072-3 (2) | - |
| 8 | HK6,8 | Hungary | | Fülöpszállás, Kelemen-szék | 46.797356 | 19.183097 | | - | | MW488074-5 (2) | - |
| 9 | HP1-3 | Hungary | | Pusztaszer, Büdös-szék | 46.546253 | 20.03282 | | OK047175-7 (3) | | MW488076-8 (3) | - |
| 10 | HS1-3 | Hungary | | Solt, Bogárzó | 46.808029 | 19.141267 | | OK047178-80 (3) | | MW488079-80 (2) | - |
| 11 | HU6-8 | Hungary | | Szabadszállás, Büdös-szék | 46.866044 | 19.169286 | | OK047181-3 (3) | | MW488081-2 (2) | - |
| 12 | HZ1-5 | Hungary | | Szabadszállás, Zab-szék | 46.837517 | 19.16978 | | OK047184-7 (4) | | MW488083 (1) | - |
| 13 | IS1-2 | Israel | | Ashdod, Tel Ashdod | 31.75361 | 34.6525 | | OK047188-9 (2) | | MW488084 (1) | - |
| 14 | M1 | Morocco | | Ifran, Daya Mertissiliouine | 33.29503 | -5.1791 | | OK047190 (1) | | MW488085 (1) | OK037209 (1) |
| 15 | SB1-5 | Serbia | | Stanišić, Bela Bara | 45.94707 | 19.090246 | | OK047191-4 (4) | | MW488086-8 (3) | - |
| **16** | **EA1-5** | **Spain** | | **Albacete, Navas del Bonillo** | **38.93533** | **-2.47305** | | **LT821337-41 (5)** | | **LT821353-7 (5)** | **-** |
| **17** | **EG1-3** | **Spain** | | **Segovia, Laguna de la Iglesia** | **41.203861** | **-4.56927** | | **LT821334-6 (3)** | | **LT821351-2 (2)** | **-** |
| 18 | 688 Tun | Tunisia | | Kelaat El Andalous, El Hisiane | 36.995056 | 10.158451 | | OK047195 (1) | | - | - |
| ***Branchinecta orientalis*** | | |  | |  | |  | |  |  |  |
| 19 | AP1-3 | Austria | | Apetlon, Apetloner Meierhoflacke | 47.721576 | 16.82404 | | OK047196-8 (3) | | MW488089 (1) | - |
| 20 | AG1-5 | Austria | | Apetlon, Grosse Neubruchlacke | 47.786121 | 16.842198 | | OK047199-203 (5) | | MW488090-1 (2) | - |
| 21 | AL1-5 | Austria | | Apetlon, Lange Lacke | 47.757475 | 16.878765 | | OK047204-8 (5) | | MW488092-4 (3) | - |
| 22 | AE1-2 | Austria | | Apetlon, Öst. Fuchslochlacke | 47.790441 | 16.866186 | | OK047209-10 (2) | | MW488095 (1) | - |
| 23 | AS1-5 | Austria | | Apetlon, Sechsmahdlacke | 47.783789 | 16.884112 | | OK047211-5 (5) | | MW488096 (1) | OK037199- 202 (4) |
| 24 | AF2-5 | Austria | | Apetlon, West. Fuchslochlacke | 47.790077 | 16.852349 | | - | | MW488097-100 (4) | - |
| 25 | AW1-4 | Austria | | Apetlon, West. Wörthenlacke | 47.770922 | 16.870777 | | OK047216-9 (4) | | MW488101-2 (2) | OK037204 (1) |
| 26 | AA1-5 | Austria | | Illmitz, Albersee | 47.775136 | 16.770119 | | OK047220-4 (5) | | MW488103-4 (2) | - |
| 27 | AK1-4 | Austria | | Illmitz, Kirchsee | 47.758679 | 16.785489 | | - | | MW488105-8 (4) | - |
| 28 | AM1-5 | Austria | | Illmitz, Mittlerer Stinkersee | 47.806759 | 16.787522 | | OK047225-9 (5) | | MW488109-10 (2) | - |
| 29 | AH1-5 | Austria | | Illmitz, Obere Höllacke | 47.827119 | 16.807932 | | OK047230-4 (5) | | MW488111 (1) | - |
| 30 | AO1-5 | Austria | | Illmitz, Oberer Stinkersee | 47.813611 | 16.792517 | | OK047235-9 (5) | | MW488112-5 (4) | - |
| 31 | AR1-5 | Austria | | Illmitz, Runde Lacke | 47.785745 | 16.792744 | | OK047240-4 (5) | | MW488116-8 (3) | - |
| 32 | AZ1-3 | Austria | | Illmitz, Zicklacke | 47.766964 | 16.784752 | | OK047245-7 (3) | | - | - |
| **33** | **CDL1-2** | **China** | | **Tibetan plateau, Dongla Pond** | **29.008611** | **90.853611** | | **MW822926-7 (3)** | | **MW829405 (1)** | **MW829399 (1)** |
| **34** | **CGQ1-3** | **China** | | **Tibetan plateau, Gangqian Lake** | **28.860278** | **90.836389** | | **MW822920, MW822925, MW822927 (10)** | | **MW829405-6 (2)** | **MW829399 (2)** |
| **35** | **CHT1-5** | **China** | | **Tibetan plateau, Huangnitang Pond** | **29.013333** | **90.935833** | | **MW822920, MW822922-4, MW822927 (11)** | | **MW829407 (2)** | **MW829399, MW829402 (3)** |
| **36** | **CMD1-2** | **China** | | **Tibetan plateau, Meiduo Lake** | **28.850833** | **91.000278** | | **MW822923 (12)** | | **MW829405 (2)** | **MW829399, MW829402 (2)** |
| **37** | **CSZ1-2** | **China** | | **Tibetan plateau, Sangzhu Lake** | **28.768611** | **90.653333** | | **MW822920, MW822927 (9)** | | **MW829407 (2)** | **MW829399 (2)** |
| **38** | **CXX1** | **China** | | **Tibetan plateau, Xiaxi Lake** | **28.889444** | **91.063333** | | **MW822916 (2)** | | **MW829407 (1)** | **-** |
| 39 | HT1-2,4 | Hungary | | Bácsalmás, Sóstó | 47.005063 | 18.491114 | | - | | MW488119-21 (3) | - |
| 40 | HB1 | Hungary | | Dunatetétlen, Böddi-szék south pool | 46.764764 | 19.147753 | | OK047248 (1) | | - | - |
| 41 | HD1 | Hungary | | Dunatetétlen, Böddi-szék southeast pool | 46.756864 | 19.156653 | | - | | MW488122 (1) | - |
| 42 | HK1-4 | Hungary | | Fülöpszállás, Kelemen-szék | 46.797356 | 19.183097 | | OK047249 (1) | | MW488123-6 (4) | OK037207-8 (2) |
| **43** | **HG1-4** | **Hungary** | | **Kiskunság, Böddi-szék** | **46.768361** | **19.147694** | | **LT821330-3 (4)** | | **LT821347-50 (4)** | **-** |
| 44 | HS6 | Hungary | | Solt, Bogárzó | 46.808029 | 19.141267 | | - | | MW488127 (1) | - |
| 45 | HU1-3 | Hungary | | Szabadszállás, Büdös-szék | 46.866044 | 19.169286 | | OK047250-2 (3) | | MW488128-30 (3) | - |
| 46 | HZ1 | Hungary | | Szabadszállás, Zab-szék | 46.837517 | 19.16978 | | - | | MW488131 (1) | - |
| 47 | MB1 | Mongolia | | Baraatiin toirom | 46.81072 | 106.29568 | | OK047253 (1) | | MW488132 (1) | OK037210 (1) |
| 48 | MK1-2 | Mongolia | | Khalzangiin goliin toirom | 47.08083 | 105.955 | | OK047254-5 (2) | | MW488133-4 (2) | OK037211 (1) |
| 49 | MS1-6 | Mongolia | | Shiliin nuuriin toirom | 47.0075 | 106.12972 | | OK047256-60 (5) | | MW488135-8 (4) | OK037212 (1) |
| **50** | **RB1** | **Russian Federation** | | **Lake Baikal, Olkhon Island** | **53.1567** | **107.3836** | | **LC469606 (1)** | | **-** | **-** |
| 51 | SO1-2,4 | Serbia | | Elemir, Okanj | 45.467165 | 20.300073 | | - | | MW488139-41 (3) | - |
| 52 | SR2-4 | Serbia | | Melenci, Mala Rusanda | 45.512444 | 20.302639 | | OK047261 (1) | | MW488142-3 (2) | - |
| 53 | SS1,3-4 | Serbia | | Novi Bečej, Slano Kopovo | 45.625224 | 20.210024 | | OK047262-4 (3) | | MW488144 (1) | - |
| 54 | SM1-2 | Serbia | | Riđica, Medura | 45.992088 | 19.133211 | | OK047265 (1) | | MW488145 (1) | - |
| 55 | ES1-5 | Spain | | Cuenca, Laguna de la Dehesilla | 39.421788 | -2.84055 | | OK047266-8 (3) | | MW488146-9 (4) | OK037206 (1) |
| **56** | **EC1-5** | **Spain** | | **Cuenca, Laguna del Hito** | **39.863944** | **-2.693361** | | **LT821325-9 (5)** | | **LT821342-6 (5)** | **-** |

Appendix B – Methods: DNA extraction, Polymerase Chain Reaction (PCR), DNA purification and sequencing

Genomic DNA was extracted from tissue using the NucleoSpin® extraction kit for individual samples (Macherey-Nagel, Düren, Germany).

To amplify the mitochondrial COI and the nuclear ITS2 DNA regions, the PCR volume of 25 μL contained 2 μL of template DNA, 12.5 μl of 2x My Taq HS mix (Bioline, London, UK) and 1 μL of primer mix (forward and reverse, 20 μM). Reaction volumes were supplemented with 9.5 μL of sterile deionized water (Sigma-Aldrich, Missouri, USA). The mitochondrial COI DNA region of *B. orientalis* and *B. ferox* was amplified using universal invertebrate forward (5’ GGTCAACAAATCATAAAGATATTGG 3’) and reverse (5’ TAAACTTCAGGGTGACCAAAAAATCA 3’) primers ^4^. When amplification was unsuccessful with these two primers, we changed the reverse primer (and combined it with the universal forward primer). Our first alternative was reverse COI-H (5’ TCAGGGTGACCAAAAAATCA 3’) primer ^5^, also used by ^3^, but eventually we also designed a new reverse primer COI_ORIENFER_R648 (5’ TGGTAAAGYATAGGATCTCCRCC 3’) for a few of the remaining samples. To amplify nuclear ITS2 DNA region, we used forward CAS5p8sFt (5’ TGAACATCGACATTTYGAACGCATAT 3’) and reverse CAS28sB1d (5’ TTCTTTTCCTCCSCTTAYTRATATGCTTAA 3’) primers ^6^. To amplify both genetic markers, the cycle settings were modified from ^7^, with an initial denaturation of 1 min at 95 °C, followed by 5 liberal amplification cycles (denaturation for 15 s at 94 °C, annealing for 15 s at 45 °C and elongation for 10 s at 72 °C) and 35 more rigid cycles (denaturation for 15 s at 95 °C, annealing for 15 s at 50 °C and elongation for 10 s at 72 °C) and a final elongation of 6 min at 72 °C.

Reaction contaminants were removed from the samples using CleanPCR beads (GC Biotech, Waddinxveen, Netherlands). Purified products were again amplified with the Big Dye Terminator 3.1 kit (Applied Biosystems, Gent, Belgium), following a 1/8 dilution of the Big Dye Terminator sequencing protocol and the corresponding primers that were specified above (3.2 μM; forward and reverse primers were used separately). The PCR program entailed one initial denaturation step of 95 °C for 1 min, followed by 30 amplification cycles (denaturation for 10 s at 95 °C, annealing for 5s at 50 °C and elongation for 4 min at 60 °C). The extension products were once again purified using the CleanDTR beads (GC Biotech, Waddinxveen, Netherlands). Finally, the products were run on an ABI PRISM 3500 Avant Genetic Analyser automated sequencer (Applied Biosystems, Gent, Belgium).

To obtain the nuclear 18S rRNA gene region, the PCR reactions were performed in 20 µL volume containing 0.8x JumpStart *Taq* ReadyMix (1 U of JumpStart Taq DNA polymerase, 20 mM KCl, 0.6 mM MgCl_2_, 4 mM Tris-HCl, 0.08 mM of dNTP; Sigma-Aldrich, Germany), 0.4 µM of both primers and about 100 ng of DNA. The region of the nuclear ribosomal 18S rRNA gene was amplified using eukaryote-specific primers complementary to the 5’-terminus (5’-TYCCTGGTTGATYYTGCCAG-3’) and the 3’-terminus (5’-TGATCCTTCCGCAGGTTCACCT-3’) ^8^ under conditions described by ^9^ but we performed PCR consisted of 35 cycles. The amplified fragments were visualized by electrophoresis on 1% agarose gel and purified by alkaline phosphatase FastAP (1 U/μl, Thermo Scientific) and exonuclease I (20 U/μl, Thermo Scientific) treatment according to the manufacturer’s guidelines. The 18S rRNA sequences were obtained from both strands using BigDyeTM sequencing protocol implemented in ABI 3730XL Genetic Analyzer (Macrogen Inc., Amsterdam, the Netherlands).

Appendix C – Phylogenetic tree based on nuclear ITS2 DNA region

Figure A1. Consensus phylogeographic tree for both *Branchinecta ferox* and *Branchinecta orientalis*, based on the nuclear ITS2 DNA region. The supporting values based on maximum likelihood and Bayesian inference are included close to the nodes. The unsupported groupings are indicated with ‘-‘.

References:

1. Deng, Z., Chen, Y., Ma, X., Hu, W. & Yin, M. Dancing on the top: phylogeography and genetic diversity of high-altitude freshwater fairy shrimps (Branchiopoda, Anostraca) with a focus on the Tibetan Plateau. *Hydrobiologia* **848**, 2611–2626 (2021).

2. Naganawa, H. *et al.* Does the dispersal of fairy shrimps (Branchiopoda, Anostraca) reflect the shifting geographical distribution of freshwaters since the late Mesozoic? *Limnology* (2019) doi:10.1007/s10201-019-00589-9.

3. Rodríguez-Flores, P. C., Jiménez-Ruiz, Y., Forró, L., Vörös, J. & García-París, M. Non-congruent geographic patterns of genetic divergence across European species of Branchinecta (Anostraca: Branchinectidae). *Hydrobiologia* **801**, 47–57 (2017).

4. Folmer, O., Black, M., Hoeh, W., Lutz, R. & Vrijenhoek, R. DNA primers for amplification of mitochondrial cytochrome c oxidase subunit I from diverse metazoan invertebrates. *Molecular marine biology and biotechnology* **3**, 294–299 (1994).

5. Machordom, A., Araujo, R., Erpenbeck, D. & Ramos, M.-Á. Phylogeography and conservation genetics of endangered European Margaritiferidae (Bivalvia: Unionoidea). *Biol J Linn Soc* **78**, 235–252 (2003).

6. Ji, Y.-J., Zhang, D.-X. & He, L.-J. Evolutionary conservation and versatility of a new set of primers for amplifying the ribosomal internal transcribed spacer regions in insects and other invertebrates. *Molecular Ecology Notes* **3**, 581–585 (2003).

7. Adamowicz, S. J., Hebert, P. D. N. & Marinone, M. C. Species diversity and endemism in the Daphnia of Argentina: a genetic investigation. *Zool. J. Linn. Soc.* **140**, 171–205 (2004).

8. Weekers, P. H. H., Murugan, G., Vanfleteren, J. R., Belk, D. & Dumont, H. J. Phylogenetic analysis of anostracans (Branchiopoda: Anostraca) inferred from nuclear 18S ribosomal DNA (18S rDNA) sequences. *Molecular Phylogenetics and Evolution* **25**, 535–544 (2002).

9. Mioduchowska, M. *et al.* Notes on genetic uniformity in the fairy shrimp Branchipus schaefferi Fischer, 1834 (Branchiopoda, Anostraca) from Poland. *North-West. J. Zool.* **14**, 127–129 (2018).
